# Occlusive bandaging of wounds with decreased circulation promotes growth of anaerobic bacteria and necrosis: case report

**DOI:** 10.1186/s13104-016-2205-1

**Published:** 2016-08-08

**Authors:** Fariba Nayeri

**Affiliations:** The Institute for Protein Environmental Affinity Surveys (PEAS Institut) and Department of Infectious Diseases, Linköping University, Linköping, Sweden

**Keywords:** Poly microbial, Occlusive bandage, Anaerobic infection, Wound care

## Abstract

**Background:**

Topical occlusive/semi-occlusive dressings that induce a damp and trapped environment are widely used in wound treatment. Subjecting the wound with impaired circulation to such trapped/air-free environment potentiates the growth of anaerobic bacteria and risk for serious infection.

**Case presentation:**

We present a case of previously healthy Swedish male that had a muscle contusion after heavy trauma that induced impaired circulation. The application of an occlusive bandage to the post-traumatic wound on the patient resulted in a poly-microbial anaerobic infection and necrosis. These complications were treated successfully with antibiotics and open dressing of the wound.

**Conclusion:**

The pathophysiology of difficult- to- treat ulcers should be reviewed by the physician and occlusive dressing should be avoided when treating wounds with impaired circulation.

## Background

Subjecting wounds to an occlusive trapped/air-free environment potentiates the growth of anaerobic bacteria, which can result in significant sepsis [1]. In a report by Mousa [2], study of 127 cases of burn wound infection revealed that 55.1 % of ulcers were infected with anaerobic bacteria. Patients with openly dressed wounds recovered more quickly from anaerobic bacterial infections than patients with occlusive wound dressings (P < 0.01). Pressure ulcers are also susceptible to infection by biofilm-growing aerobic and anaerobic bacteria, the biofilm formation on the wound being the main reason for its delayed healing [3]. Survey of bacterial diversity in chronic wounds using pyrosequencing [4] showed that 62 % of the bacterial populations in pressure ulcers were identified as obligate anaerobes.

The present case report describes a post-traumatic wound complicated with polymicrobial anaerobic infection and necrosis. We aim to emphasize the importance of the assessment of pathophysiology of wounds in order to gain a better understanding of the wound’s microbiota and recommendation of appropriate wound dressings by the physician before their routine application by medical assistants.

## Case presentation

**Fig. 1:**
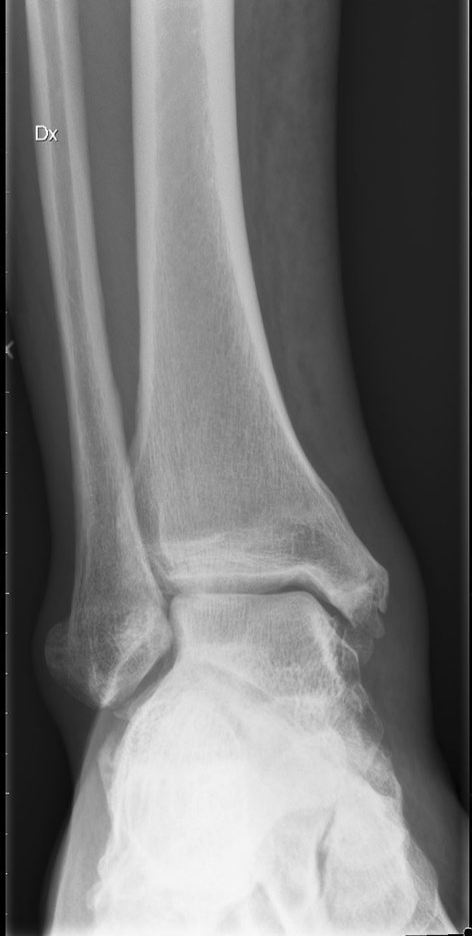
This X-ray image from the right leg and wrist from the patient presented in the case report is taken on 13th April

A previously healthy and active 53-year-old Swede male who worked full-time in the metal industry had arthrosis of the right knee and had experienced episodes of pain in his right ear since 2011. The patient was injured when a 400-kg metal device fell on his right leg during the course of his work (13th March). The patient was wearing security shoes and clothes at the time of the accident. He presented with a superficial abrasion (15 cm × 3 cm) on the front of the right lower leg, without heavy bleeding, and swelling of the right ankle. X-rays and blood tests ruled out a fracture and organ failure (Fig. 1). He was assessed by the attending surgeon at the University hospital in Linköping. Surgical intervention or revisions were not indicated and the patient was dismissed with a recommendation for local antiseptics and elevation of the leg while sitting or lying down.

**Fig. 2:**
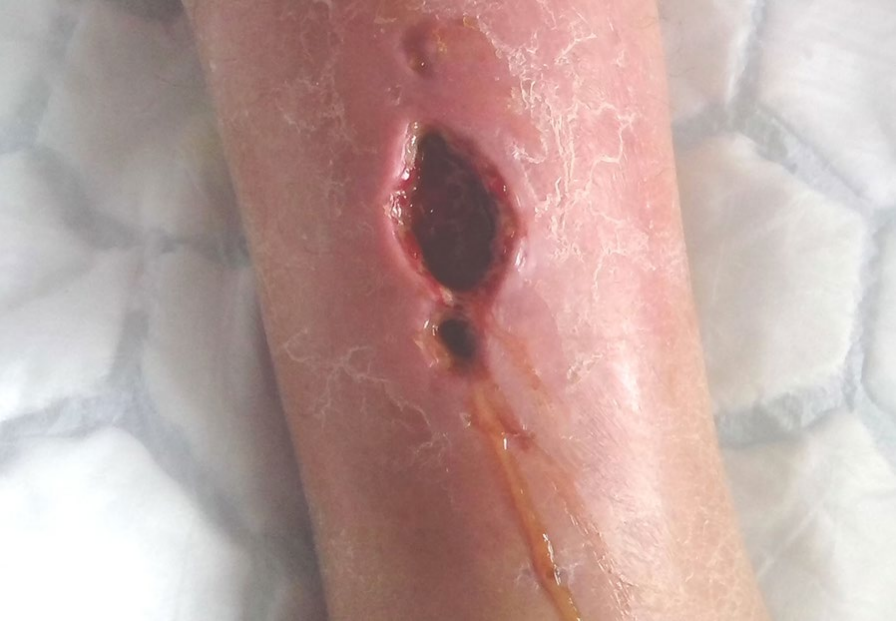
The infected post-traumatic wound with necrosis and odorous secretion, 14 April 2015

A nursing assistant at the Occupational Health Center cared for the patient’s abrasion; it was covered regularly during 4 weeks every other day with an occlusive bandage (Mepilex foam dressing, Mölnlycke Health Care, Sweden), a wound dressing material that is routinely used at health centers. The patient had no fever during this period and did not change the dressing himself. There is no document about him contacting the Department of Infectious Diseases or the health center during this period. He had noticed that the ulcer was producing odorous discharge, but the attending nurse did not experience a situation that should be referred to specialist. The patient was referred to the hospital (13th April) due to visible muscle necrosis accompanied by yellow, odorous secretion at the bottom of an ulcer on the front of the right leg (Fig. 2). The patient had no fever, and his vital parameters were stable, although he did have diffuse redness and pitting edema on the right leg. The laboratory analysis revealed normal white blood cells, creatinine, electrolytes, and c-reactive protein 30 (<5 mg/l).

The local status motivated an ulcer revision and debridement of muscle tissue necrosis. However, there was no sign of acute inflammation in the ulcer area and with respect to the severe necrosis and significant growth of anaerobic bacteria together with gram-positive bacteria in a biofilm [5, 6], there was a risk of developing a larger ulcer area with impaired healing. The clinical status of patient was stable. Intravenous meropenem (3 × 1 g) was started immediately after cultures from blood and ulcer secretion were secured. A culture from the ulcer secretion revealed growth of *Staphylococcus aureus*, *Streptococcus beta hemolytic group G*, *Clostridium innocuum*, and *Bacterioides thetaiotaomicron*. The ulcer was treated conservatively with the local application of an antibiotic gel containing 250 mg vancomycin and hepatocyte growth factor (HGF in 100 IU antithrombin III Baxter) [7] plus sodium chloride for 2 days, followed by antithrombin III plus sodium chloride gel for 5 days. The wound dressing comprised sterile cotton compresses that were changed daily during the first week.

**Fig. 3:**
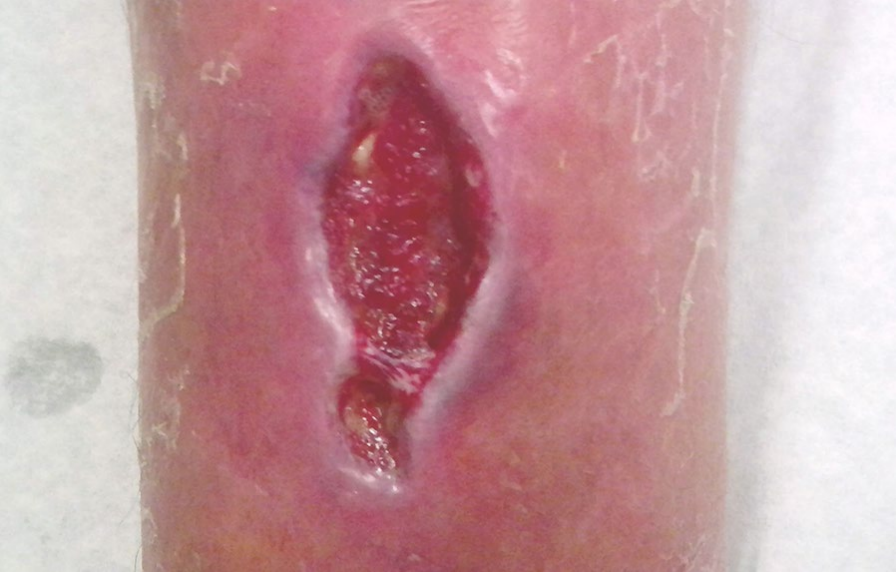
After 10 days of treatment, the ulcer shows signs of granulation and fresh bleeding

**Fig. 4:**
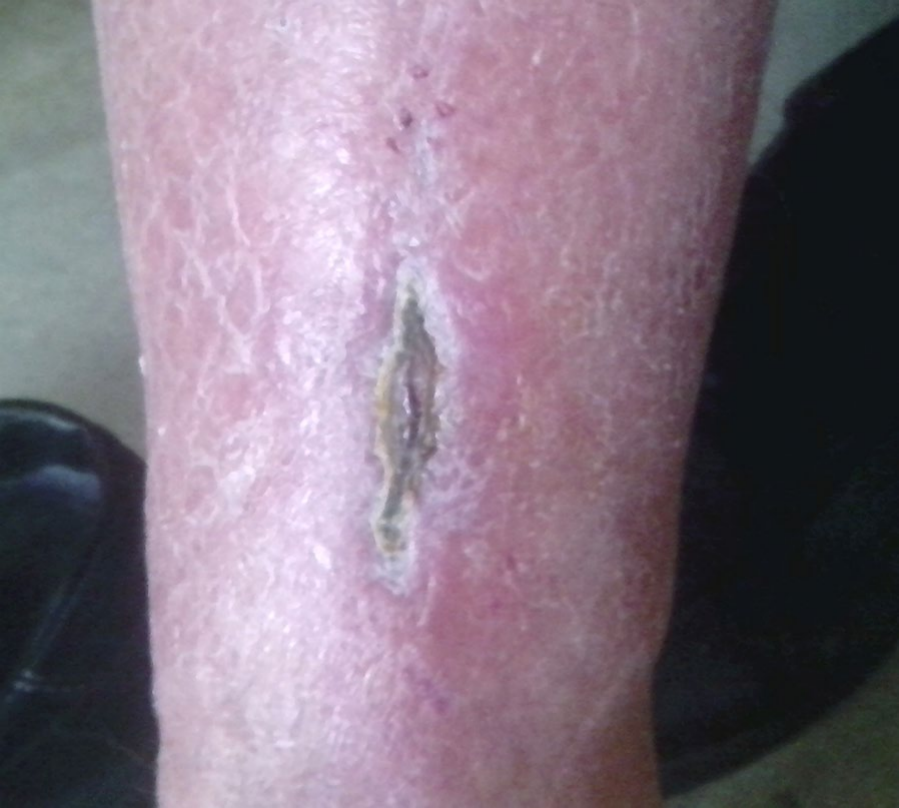
Last control, 1 June 2015

The first sign of fresh bleeding was observed within 1 week (Fig. 3), and the patient was dismissed with oral amoxicillin + clavulanacid and metronidazole 3 × 500 mg. The patient taught himself to dress the ulcer at home with sterile cotton compresses and attended regular follow-ups at the clinic once each week. Antibiotic therapy was ceased after 20 days of treatment, and the patient returned to full-time work within 4 weeks. The follow-up controls showed complete healing of ulcer (Fig. 4).

## Discussion

Anaerobic bacteria are a common cause of infections, some of which can be serious and life-threatening [8]. Because of their fastidious nature, anaerobes are hard to isolate and are often not recovered from infected sites. The delayed or inappropriate therapy against these organisms may lead to failures in eradication of these infections [9]. *Clostridium innocuum* is a relatively antimicrobial-resistant, frequently misidentified anaerobe that has been associated with bacteremia [10]. Clostridial myonecrosis is a rapidly progressive disease characterized by muscle necrosis and systemic toxicity [11, 12]. In a 1997 report, Mousa found that *Bacteroides* species were isolated from 14 of 17 cases with burn wounds that developed septic shock, and were also recovered from four patients who had anaerobic infection alone [2].

The patient in the present case had a muscle contusion after heavy trauma that induced impaired circulation and was susceptible to a trapped, air-free environment under an occlusive dressing. The discolored subcutaneous tissue, odorous secretion, slightly increased acute phase proteins, and growth of *S. aureus*, *Streptococcus beta hemolytic group G*, *C. innocuum*, and *B. thetaiotaomicron* might indicate a serious infection and risk for deleterious consequences. However, because of the patient’s immune competence and appropriate antibiotic therapy, he survived this life-threatening situation.

## Conclusion

Wounds with impaired circulation are highly vulnerable to infection. Anaerobic and gram-positive bacteria are over-presenting microorganisms in development of biofilms in such wounds. Occlusive dressing should be avoided when treating wounds with impaired circulation such as after heavy trauma, burn ulcers or pressure ulcers. The conventional open clean cotton dressings are recommended in such cases.

## Patient consent

Written informed consent was obtained from the patient for publication of this case report and any accompanying images.
